# Trends in emergency department visits for acute intoxication: a 3-year retrospective study in Hangzhou, East China

**DOI:** 10.3389/fpubh.2025.1598559

**Published:** 2025-06-27

**Authors:** Chengle Li, Hong Zhang, Bo Yang

**Affiliations:** ^1^Department of Pharmacy, Tongde Hospital of Zhejiang Province, Hangzhou, China; ^2^Zhejiang Academy of Traditional Chinese Medicine, Hangzhou, China; ^3^Department of Functional Examination, Tongde Hospital of Zhejiang Province, Hangzhou, China

**Keywords:** acute intoxications, overdose, suicide, emergency department, hospitalization

## Abstract

**Background:**

Epidemiological studies on acute intoxication contribute to the development of emergency care, particularly in hospital settings. Understanding the demographics and risk factors of acute intoxication aids in designing targeted public health interventions and educational campaigns to reduce its incidence.

**Objective:**

The objective of this research was to describe the epidemiological trends and patterns of acute intoxication cases, as well as the types of intoxication, at Tongde Hospital of Zhejiang Province, a general hospital located in eastern China.

**Methods:**

A retrospective review of medical records was conducted for all patients presented to the emergency department with acute intoxication at Tongde Hospital of Zhejiang Province between 2020 and 2022. The data collected from the hospital information system included gender, age, visit time, types of intoxication cases, length of hospital stay, treatment administered, and clinical outcomes. Univariate analysis was performed to identify factors associated with suicidal and non-suicidal patients.

**Results:**

During the three-year period from 2020 to 2022, a total of 550 acute intoxication patients were admitted to the emergency department. Females constituted the majority of the cohort, representing 72.3% (*n* = 398) of the total cases, and the age group of 13 to 40 years made up the largest proportion at 74.7%. The number of cases increased annually, with poisoning incidents varying by month and week. The most common substances involved were drugs (79.6%), followed by alcohol mixed with drugs (5.8%), gases (6.2%), pesticides (3.8%), household chemical products (3.1%), and plants (1.4%). The monthly distribution of cases showed peaks in June, September, and April, while the weekly distribution peaked on Mondays and Thursdays. The mean time interval between toxin exposure and hospital admission was 5.40 ± 9.93 h. Statistical analysis revealed that gender, age, and the type of poisonous substance were all significant factors associated with suicide. After treatment, 80.5% (*n* = 443) of patients were discharged home within 24 h, while 19.5% (*n* = 107) required hospitalization.

**Conclusion:**

This study indicates an increasing proportion of suicidal cases among all poisoning incidents, particularly among young women. Drug intoxication was the most common cause, with antidepressants, antipsychotics, and benzodiazepines being the most commonly involved substances. These findings underscore the urgent need for mental health support, pharmaceutical safety measures, and preventive education targeting high-risk groups, particularly young women.

## 1 Introduction

Acute poisoning is defined as the adverse effects caused by a single or short-term exposure to a toxic substance. This can occur through various routes of exposure, including ingestion, inhalation, dermal contact, or injection, and represents a common reason for admission to hospital emergency departments and intensive care units (ICUs) (1). Acute poisoning may be intentional, often as a means of self-harm or suicide attempt, or unintentional, such as accidental or occupational exposure.

Suicide-related poisoning remains a significant global public health issue. According to the World Health Organization, more than 700,000 people die by suicide annually, with many more attempting it (2). In the United States, suicide ranks as the fourth leading cause of death among individuals aged 15–29, with poisoning being the third most commonly used method (3, 4). In China, poisoning and related injuries rank as the fifth leading cause of mortality, based on findings from the Third National Cause-of-Death Survey (5). Despite this burden, comprehensive epidemiological data on acute poisoning in China remain limited, particularly regarding regional variations and temporal trends.

Significant global and regional disparities exist in the causes of acute poisoning. Pesticide poisoning remains the predominant form in low-and middle-income countries, while in high-income countries, poisoning with household products and medications was more prevalent (6, 7). A retrospective analysis of 95,754 acute poisoning cases reported to China’s Health Emergency Information Platform between 2016 to 2022 found that pesticide poisoning accounted for 30.4% of all cases (8). However, recent data from our hospital indicate a different pattern, with drug poisoning being the most common type—contrary to previous reports in the literature. To better understand the current epidemiological profile of acute poisoning at our institution, we conducted this study.

This research was conducted at Tongde Hospital in Hangzhou, a major city in eastern China. Located in the western part of Hangzhou, Tongde Hospital is a strategic location for studying trends in acute poisoning and their underlying factors. The aim of this study was to analyze the epidemiological trends and characteristics of acute intoxication cases over a three-year period (2020–2022) in this setting. These findings contribute to addressing the gap in epidemiological data on poisoning in major eastern cities of China. The observed trends can facilitate the improvement of surveillance and early warning systems for poisoning incidents, and support the development of targeted prevention strategies tailored to specific populations, time periods, and types of toxic substances.

## 2 Patients and methods

### 2.1 Study design and setting

A retrospective analysis was conducted on patients diagnosed with acute poisoning admitted to the emergency department of Tongde Hospital in Zhejiang Province from January 1, 2020, to December 31, 2022. Tongde Hospital is one of the largest general hospitals in western Hangzhou and hosts the Zhejiang Mental Health Center, which plays a key role in the management of mental health disorders. The hospital includes specialized departments such as the ICUs, psychiatry, and psychosomatic medicine, enabling direct admission of patients with depression-related suicide attempts without the need for transfer to other facilities. This study was approved by the Ethics Committee of Zhejiang Provincial Hospital (Approval Number: PRO2025-150-JY). Given the retrospective nature of this study, the committee waived the requirement for informed consent.

### 2.2 Selection of study patients

The study population included all patients who presented to the emergency department with overdose or accidental and intentional acute poisoning between 2020 and 2022. Patients were excluded if they had solely alcohol intoxication, animal bites, or concomitant acute or life-threatening medical conditions. Cases with incomplete medical records were also excluded. A total of 2,227 patients with acute poisoning were admitted to the hospital, among which 550 patients met the inclusion criteria after excluding 1,677 cases that did not meet the criteria, including those with simple acute alcohol intoxication.

### 2.3 Data collection

A retrospective analysis was used to collect characteristic data from all enrolled patients. The following data were collected: age, sex, time from poisoning to rescue; clinical data included type of poison, mode of poisoning, unintentional or intentional purpose, week and month of poisoning, hospitalization status, length of hospitalization, type of treatment, and prognosis. This was carried out by clinicians (HZ) and pharmacists (CL) through the review and documentation of electronic medical records.

To determine the toxicological profile of patients of different ages and genders, patients were categorized into six age groups: infants and preschoolers (1–5), school children (6–12), teenagers (13–19), young adults (20–40), old adults (41–65), and older adults (>65). Poisons were classified into six main groups: drugs, alcohol-mixed drugs, pesticides, gases, food, and chemicals. The monthly and weekly distributions of different intoxication cases were also investigated.

### 2.4 Outcomes

The main outcomes were to observe and analyze the epidemiological trends of acute poisoning cases admitted to our hospital, including the characteristics of different time periods, age distribution, gender differences, and types of poisoning; and to evaluate the treatment of patients with acute poisoning, and to explore whether there is a correlation between the date, age, gender, and type of poisoning.

### 2.5 Statistical analysis

Data were statistically analyzed using SPSS software (version 26.0; IBM Corp; Chicago, United States). Count data were expressed as absolute numbers and percentages. *χ*^2^ test was used to assess the association between poisoning and the factors of date, sex, and poisoning type. When the assumptions for the chi-square test were not met, Fisher’s exact test or its modified version was applied. Measurement data were first assessed for normality. Normally distributed continuous variables were summarized as mean ± standard deviation (SD) and analyzed using independent *t*-tests. For non-normally distributed data such as age were presented as medians with interquartile ranges [M (Q1, Q3)]. A *p*-value of less than 0.05 was considered statistically significant.

## 3 Results

**Figure 1:**
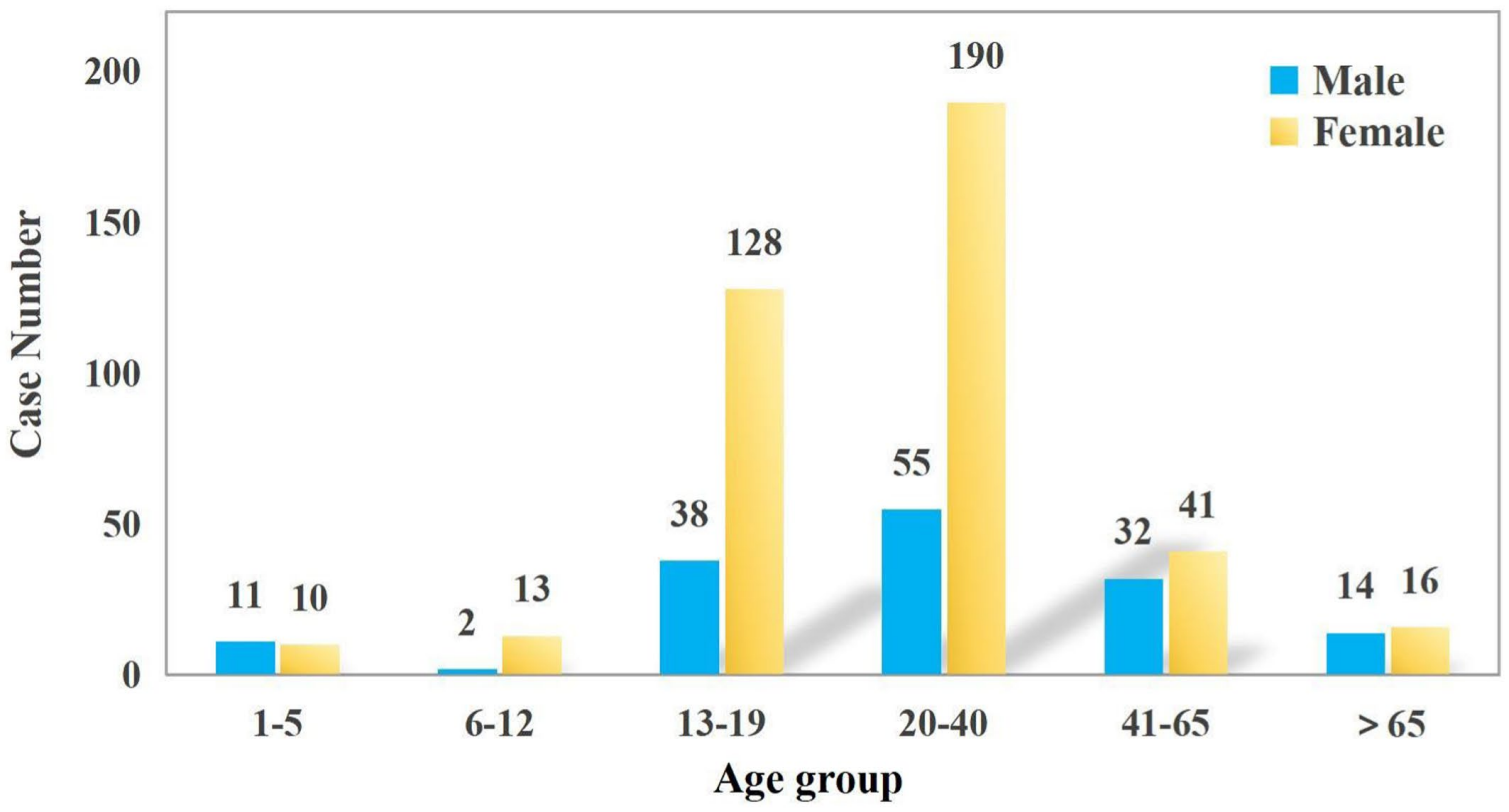
Age distribution of patients with acute poisoning.

**Figure 2:**
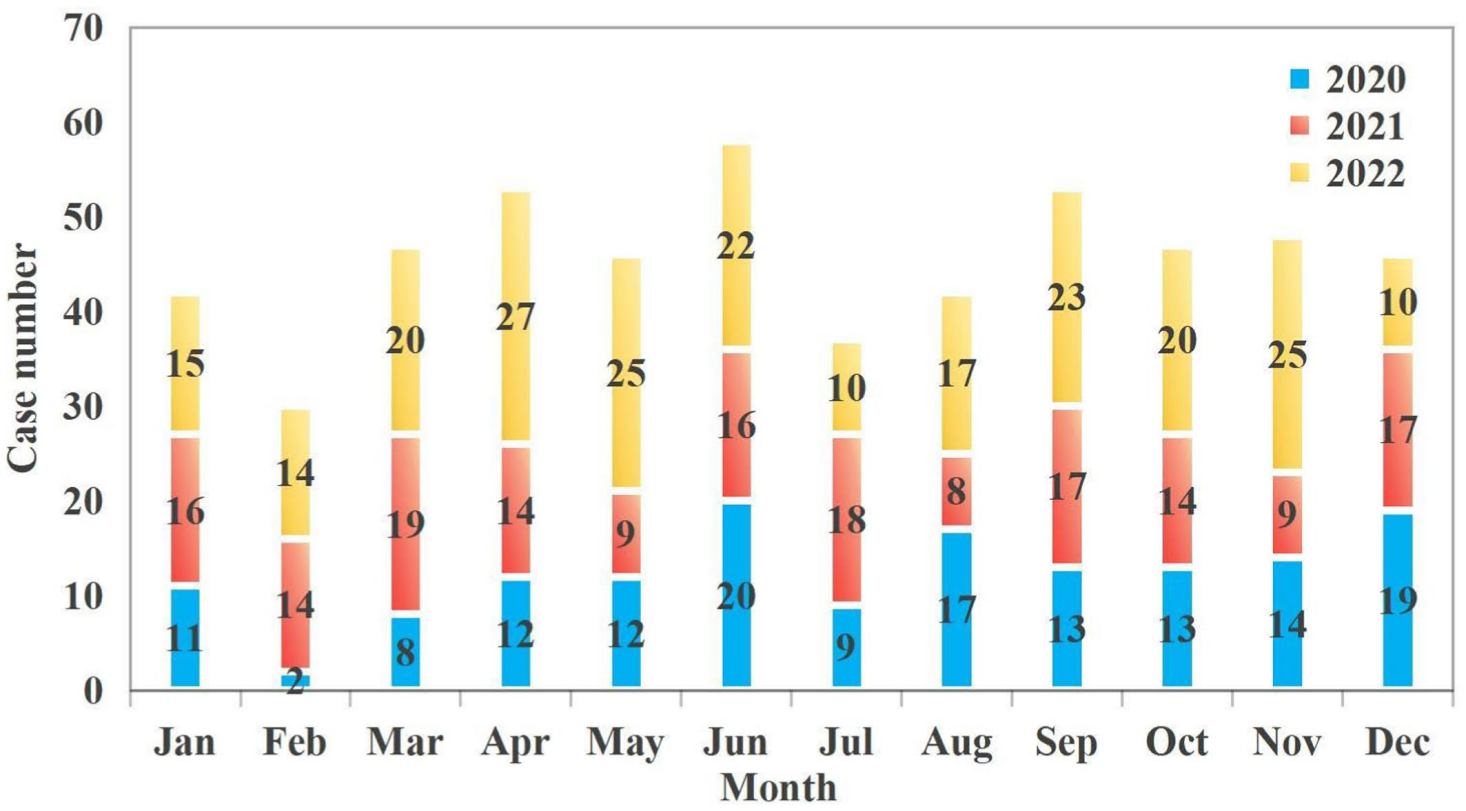
Month distribution of patients with acute poisoning.

**Figure 3:**
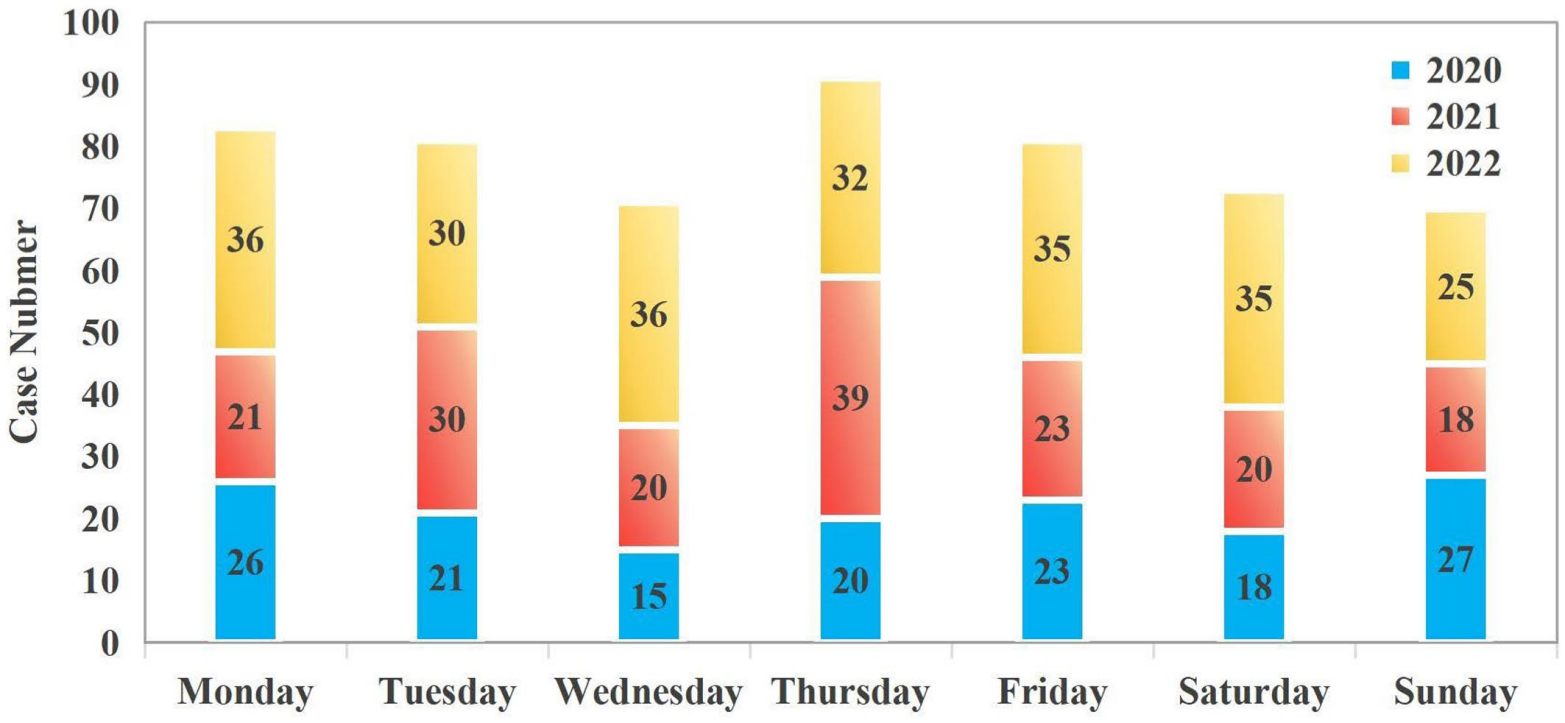
Week distribution of patients with acute poisoning.

**Figure 4:**
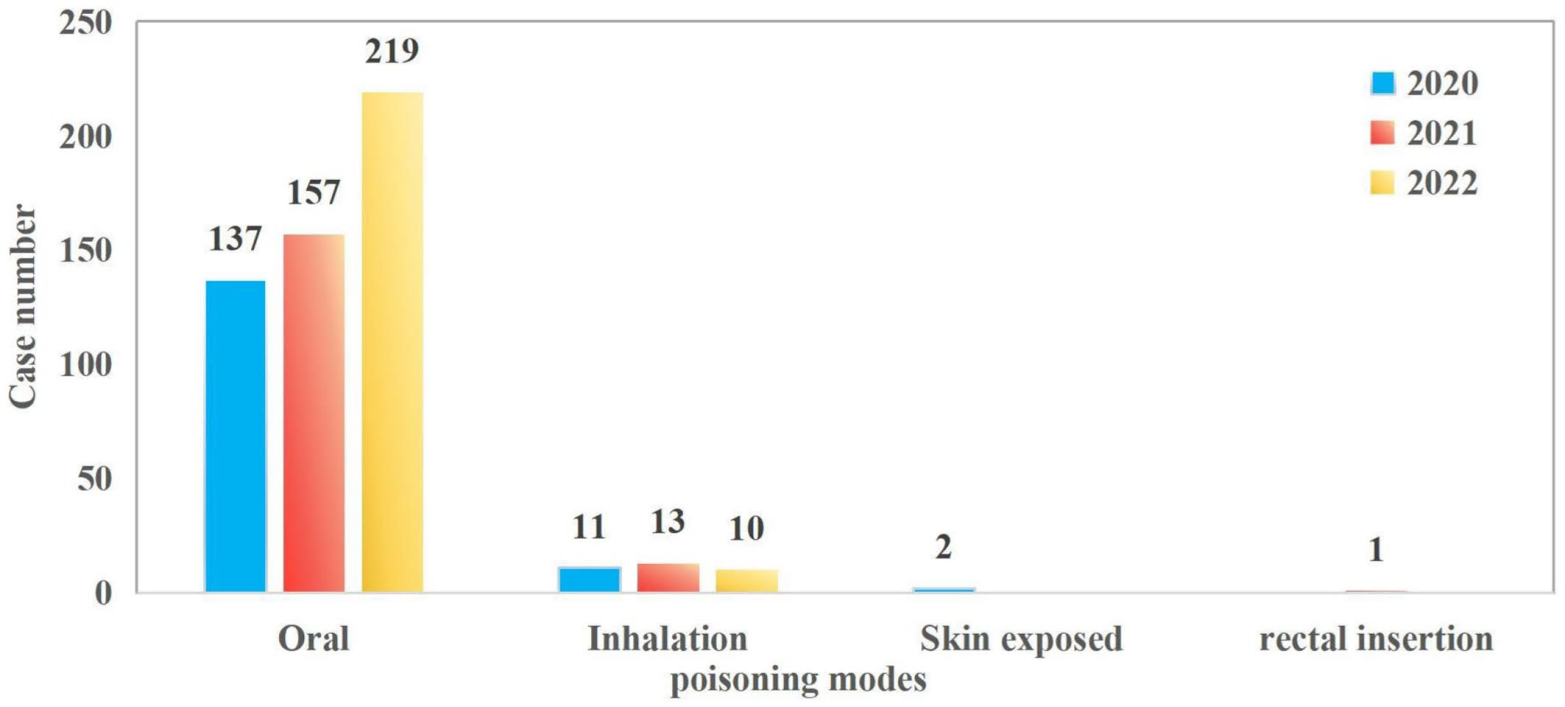
Poisoning modes distribution of patients with acute poisoning.

The study included a total of 550 patients. The median age was 23 years (IQR: 16–34). The proportion of females was significantly higher than that of males in the 13–19 and 20–40 age groups (23.27% vs. 6.91 and 34.55% vs. 10.00%, respectively). Women within these age groups are more vulnerable to poisoning incidents, which may be attributed to a higher incidence of psychological stress, emotional distress, or suicidal behavior. In contrast, males slightly outnumbered females in the youngest age cohort (1–5 years), though the difference in proportional representation was marginal. This may be due to the fact that poisoning in this age group is mostly due to accidental factors such as accidental ingestion (Figure 1). The number of patients seeking emergency care increased annually, from 150 cases in 2020 to 171 in 2021 and 229 in 2022. Poisoning cases have shown a year-on-year increasing trend, with the most significant rise occurring in 2022. This may be related to the increased prevalence of mental health issues and social stress following the epidemic. Poisoning incidence also varied by month and day of the week. Peaks were observed in June (10.5%), September (9.6%), and April (9.6%), whereas the lowest rates occurred in February (5.5%), July (6.7%), January (7.6%), and August (7.6%) (Figure 2). Weekly trends showed fewer cases on weekends—Saturdays (13.3%) and Sundays (12.7%)—compared to Mondays (15.1%) and Thursdays (16.5%) (Figure 3). Intentional poisoning (suicidal intent) was the most common cause, accounting for 434 cases (78.9%). Oral ingestion was the predominant route of exposure, reported in 513 cases (93.27%). Inhalation was involved in 34 cases, dermal exposure in 2 cases, and rectal insertion in 1 case (Figure 4).

### 3.1 Toxic related information

The toxic substances were categorized into six groups: drugs, alcohol-mixed drugs, pesticides, gases, plants, and household chemical products. Significant differences in poisoning types were observed by gender and age (*p* < 0.001). Females were more commonly affected in drug poisoning cases, making up 79.0% (346/438) of all such incidents. Among female patients, benzodiazepines were the most common agent, accounting for 75.5% (71/94) of reported cases. In contrast, males were predominantly affected by chemical poisoning (82.4%, 14/17), pesticide poisoning (57.1%, 12/21), and all methanol poisoning cases.

The highest frequency of drug poisoning occurred in the 13–19 age group (35.6%, 156/438), with antidepressants representing the main cause among adolescents (61.3%, 57/93). Alcohol-drug poisoning was mainly seen in young adults aged 20–40 years (68.8%, 22/32). Antihistamine poisoning was most common in children under 5 years old (55.6%, 5/9), suggesting a high risk of accidental exposure in this group. Plant-based and organophosphorus pesticide poisonings were more prevalent among individuals over 65 years, likely due to consumption of wild vegetables and involvement in agricultural activities. Overall, drug-related poisoning was most common among adolescents and young adults, showing significant age-related variation (*p* < 0.001). In contrast, no significant age differences were found for other poisoning types (*p* > 0.05) (Table 1).

**Table 1:** Distribution of poisonous substances, gender and age.

Category	Total cases	Gender, *n* (%)		*p*-value	Age groups, *n* (%)						*p*-value
		Male	Female		Infants	School children	Teenagers	Young adults	Old adults	Older adults	
				<0.001^a^	≤5	6–12	13–19	20–40	41–65	>65	<0.001^a^
Drugs	438	92 (21.0)	346 (79.0)	>0.05^a^	15 (3.4)	14 (3.2)	156 (35.6)	182 (41.6)	50 (11.4)	21 (4.8)	<0.001^b^
Antipsychotics	88	14 (15.9)	74 (84.1)		1 (1.1)	6 (6.8)	30 (34.1)	39 (44.3)	7 (8.0)	5 (5.7)	
Antidepressant	93	22 (23.7)	71 (76.3)		0 (0.0)	1 (1.1)	57 (61.3)	13 (14.0)	18 (19.4)	4 (4.3)	
Benzodiazepine	94	23 (24.5)	71 (75.5)		0 (0.0)	1 (1.1)	13 (13.8)	63 (67.0)	10 (10.6)	7 (7.4)	
Antipsychotics + antidepressant	53	11 (20.8)	42 (79.2)		2 (3.8)	3 (5.7)	26 (49.1)	17 (32.1)	4 (7.5)	1 (1.9)	
Antipyretics	21	5 (23.8)	16 (76.2)		4 (19.0)	1 (4.8)	9 (42.9)	6 (28.6)	1 (4.8)	0 (0.0)	
Antihypertensive	11	1 (9.1)	10 (90.9)		1 (9.1)	0 (0.0)	5 (45.5)	1 (9.1)	3 (27.3)	1 (9.1)	
Antibiotics	16	3 (18.8)	13 (81.3)		1 (6.3)	0 (0.0)	4 (25.0)	10 (62.5)	0 (0.0)	1 (6.3)	
Antihistamine	9	3 (33.3)	6 (66.7)		5 (55.6)	0 (0.0)	4 (44.4)	0 (0.0)	0 (0.0)	0 (0.0)	
Others	53	10 (18.9)	43 (81.1)		1 (1.9)	2 (3.8)	8 (15.1)	33 (62.3)	7 (13.2)	2 (3.8)	
Alcohol + drugs	32	12 (37.5)	20 (62.5)	>0.05^a^	0 (0.0)	0 (0.0)	4 (12.5)	22 (68.8)	6 (18.8)	0 (0.0)	>0.05^b^
Alcohol + antibiotics	7	4 (57.1)	3 (42.9)		0 (0.0)	0 (0.0)	2 (28.6)	4 (57.1)	1 (14.3)	0 (0.0)	
Alcohol + psychotropic drugs	6	3 (50.0)	3 (50.0)		0 (0.0)	0 (0.0)	1 (16.7)	3 (50.0)	2 (33.3)	0 (0.0)	
Alcohol + others	19	5 (26.3)	14 (73.7)		0 (0.0)	0 (0.0)	2 (10.5)	14 (73.7)	3 (15.8)	0 (0.0)	
Pesticides	21	12 (57.1)	9 (42.9)	>0.05^b^	1 (100)	0 (0.0)	0 (0.0)	9 (42.9)	7 (33.3)	3 (14.3)	
Organophosphate	10	8 (80.0)	2 (20.0)		0 (0.0)	0 (0.0)	0 (0.0)	4 (40.0)	3 (30.0)	3 (30.0)	
Rat poison	2	0 (0.0)	2 (100.0)		0 (0.0)	0 (0.0)	0 (0.0)	1 (50.0)	1 (50.0)	0 (0.0)	
Herbicides	2	1 (50.0)	1 (50.0)		0 (0.0)	0 (0.0)	0 (0.0)	1 (50.0)	1 (50.0)	0 (0.0)	
Pyrethroids	5	3 (60.0)	2 (40.0)		1 (20.0)	0 (0.0)	0 (0.0)	1 (20.0)	2 (40.0)	0 (0.0)	
Others	2	0 (0.0)	2 (100.0)		0 (0.0)	0 (0.0)	0 (0.0)	2 (100.0)	0 (0.0)	0 (0.0)	
Gases	34	19 (55.9)	15 (44.1)	>0.05^b^	1 (2.9)	0 (0.0)	1 (2.9)	24 (70.6)	7 (20.6)	1 (2.9)	>0.05^b^
CO	33	18 (54.5)	15 (45.5)		1 (3.0)	0 (0.0)	0 (0.0)	24 (72.7)	7 (21.2)	1 (3.0)	
Chlorine	1	1 (100.0)	0 (0.0)		0 (0.0)	0 (0.0)	1 (100.0)	0 (0.0)	0 (0.0)	0 (0.0)	
Plant	8	3 (37.5)	5 (62.5)	>0.05^b^	0 (0.0)	0 (0.0)	2 (25.0)	3 (37.5)	0 (0.0)	3 (37.5)	>0.05^b^
Wild fruit	3	1 (33.3)	2 (66.6)		0 (0.0)	0 (0.0)	2 (66.6)	0 (0.0)	0 (0.0)	1 (33.3)	
Fungus	2	1 (50.0)	1 (50.0)		0 (0.0)	0 (0.0)	0 (0.0)	2 (100.0)	0 (0.0)	0 (0.0)	
Pickled plant (nitrite)	3	1 (33.3)	2 (66.6)		0 (0.0)	0 (0.0)	0 (0.0)	1 (33.3)	0 (0.0)	2 (66.6)	
Chemical	17	14 (84.2)	3 (17.6)	>0.05^b^	4 (23.5)	1 (5.9)	3 (17.6)	5 (29.4)	2 (11.8)	2 (11.8)	>0.05^b^
Methanol	4	4 (100)	0 (0.0)		0 (0.0)	0 (0.0)	0 (0.0)	3 (75)	1 (25)	0 (0.0)	
Disinfectant	6	4 (66.7)	2 (33.3)		2 (33.3)	1 (16.7)	1 (16.7)	1 (16.7)	1 (16.7)	0 (0.0)	
Others	7	6 (85.7)	1 (14.3)		2 (28.6)	0 (0.0)	2 (28.6)	1 (14.3)	0 (0.0)	2 (28.6)	

a Pearson chi square test.

b Fisher exact.

### 3.2 Analysis of clinical data of patients admitted with poisoning

**Figure 5:**
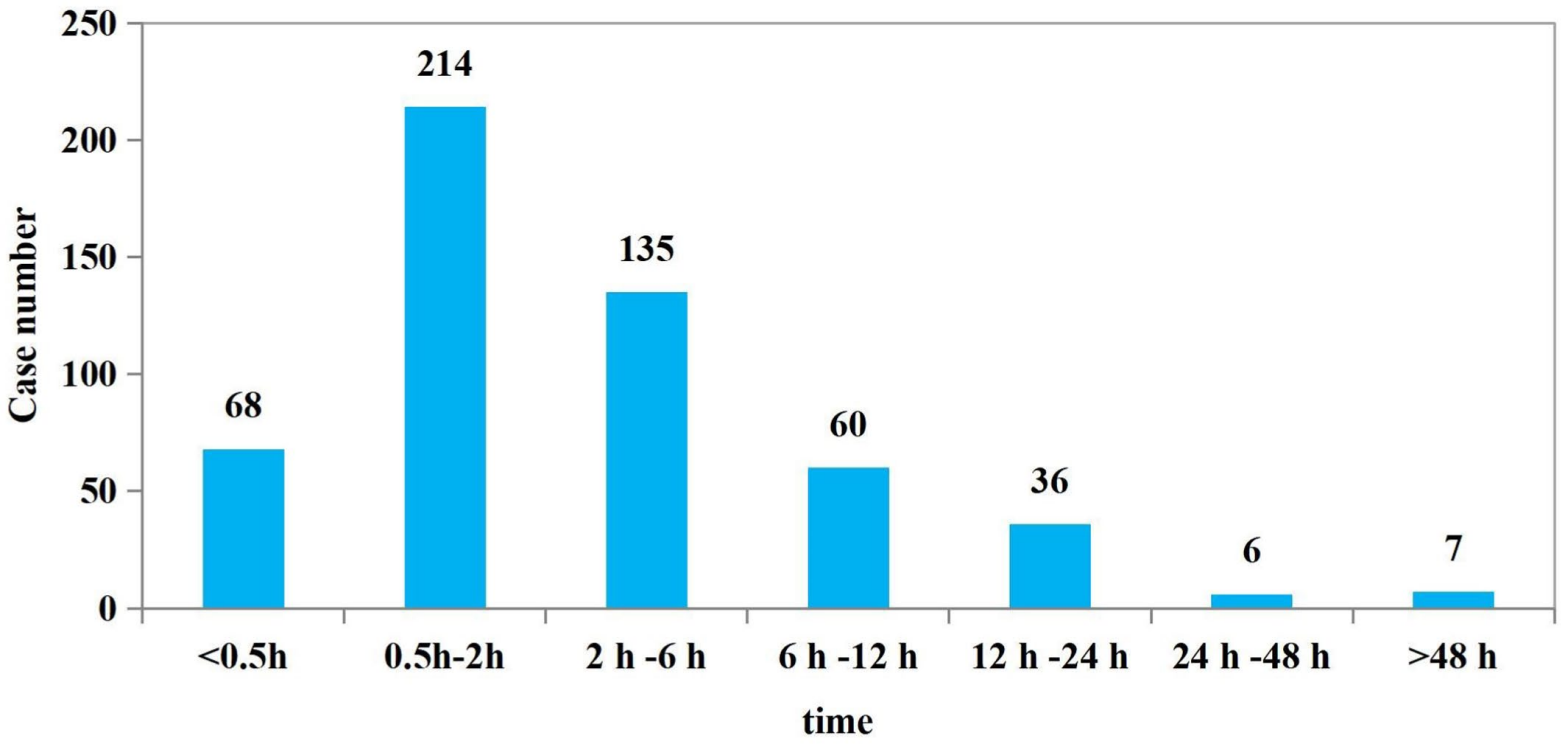
Time from poisoning to admitting in the emergency department.

A total of 526 patients had documented time intervals from poisoning to hospital visit, with a mean time of 5.40 ± 9.93 h. Among these, 417 patients (79.3%) sought medical care within 6 h of exposure (Figure 5). About 20.7% of patients still sought medical attention more than 6 h after poisoning, which may affect the effectiveness of treatment, especially for certain poisons with rapid toxic effects (e.g., organophosphorus pesticides, methanol, etc.). A total of 443 patients (80.5%) discharged from the emergency resuscitation room within 24 h after treatment. While107 patients (19.5%) required hospitalization. Of those hospitalized, 90 were admitted to the emergency intensive care units (ICUs); with a median length of stay in the ICUs was 3 days (IQR: 2–6). Seventeen patients were transferred to the psychiatric ward after resuscitation, with a median inpatient stay of 23 days (IQR: 13.5–29.5) (Table 2). The results showed that most of the poisoning cases were mild or moderate and could be stabilized after initial detoxification, gastric lavage and supportive treatment. A small number of patients were in serious condition and required further hospitalization. Poisoned patients with underlying mental health issues or suicidal intent tend to have significantly longer hospital stays. This not only reflects the increased demand for medical resources associated with psychological factors, but also highlights the greater complexity involved in treating this patient population.

**Table 2:** Statistical characteristics of hospitalized patients with poisoning.

Variable	*n* (percentage %)	Admission department	
Admission department		ICUs	Psychiatry
		90 (84.1)	17 (15.9)
Male	31 (29.0)	27 (25.2)	4 (3.7)
Female	76 (71.0)	63 (58.9)	13 (12.1)
Age (years)			
≤5	4 (3.7)	4 (3.7)	0 (0)
6–12	4 (3.7)	4 (3.7)	0 (0)
13–19	32 (29.9)	24 (22.4)	8 (7.5)
20–40	35 (32.7)	32 (29.9)	3 (2.8)
41–65	20 (18.7)	15 (14.0)	5 (4.6)
>65	12 (11.2)	11 (10.3)	1 (0.9)
Cause of poisoning			
Suicidal	87 (81.3)	72 (67.3)	15 (14.0)
Accidental	20 (18.7)	18 (16.8)	2 (1.9)
Route of exposure			
Oral	96 (89.7)	79 (73.8)	17 (15.9)
Inhalation	9 (8.41)	9 (8.41)	0 (0)
Skin exposed	2 (1.87)	22 (1.87)	0 (0)
Length of stay			
<2	29 (27.1)	29 (27.1)	0 (0)
3–5	32 (29.9)	32 (29.9)	0 (0)
6–10	24 (22.4)	22 (20.6)	2 (1.9)
11–20	10 (9.3)	5 (4.7)	5 (4.7)
>20	12 (11.2)	2 (1.9)	10 (9.3)

### 3.3 Univariate analysis of factors associated with suicide

A univariate analysis was conducted to compare clinical data between patients with and without suicidal intent, aiming to identify potential risk factors associated with suicide attempts. Suicidal intent was identified in 78.9% of all poisoning cases, with a significantly higher proportion observed among female patients (84.7%) compared to male patients (63.8%) (*p* < 0.001). Patients aged 13–19 and 20–40 years exhibited significantly higher rates of suicidal behavior than those in other age groups (*p* < 0.001). Poisoning incidents involving gases, chemicals, and plants were generally due to accidental exposure, while those involving drugs and pesticides were more often related to suicidal intent. The statistical results indicated that age, gender, and the type of toxic substance were all significantly linked to suicidal behavior (*p* < 0.001) (Table 3).

**Table 3:** Univariate analysis of factors associated with suicide.

Factor	Suicide	Non-suicide	*p*-value
Gender			<0.001^a^
Male	97	55	
Female	337	61	
Age			<0.001^a^
≤5	0	21	
6–12	12	3	
13–19	148	18	
20–40	205	40	
41–65	51	22	
>65	12	18	
Substance			<0.001^a^
Drugs	384	54	
Alcohol + drugs	24	8	
Pesticides	19	2	
Gases	5	29	
Others (plant, chemical)	2	23	

a Pearson chi square test.

### 3.4 Treatments

A total of 63 antidote administrations were recorded, including the use of atropine, acetylcysteine, flumazenil, pralidoxime iodide, neostigmine, methylene blue, and sodium dimercaptosulphonate (Table 4). Specific antidotes were administered based on the type of poisoning: flumazenil for benzodiazepine poisoning, atropine for organophosphate pesticide poisoning, neostigmine for scopolamine poisoning, methylene blue for nitrite poisoning, and sodium dimercaptopropane sulfonate for heavy metal poisoning. Seven patients underwent blood purification therapy, including two cases of lithium carbonate poisoning, three of pesticide poisoning, one of antidepressant poisoning, and one of carbon monoxide poisoning.

**Table 4:** Use of antidotes and blood purification.

Variable	Frequency	Percentage (%)
Antidotes		
Flumazenil	32	5.82%
Sodium dimercaptosulphonate	2	0.36%
Atropine	7	1.27%
Pralidoxime iodide	4	0.73%
Acetylcysteine	6	1.09%
Neostigmine	3	0.55%
Methylene blue	2	0.36%
Blood purification cases		
Lithium carbonate poisoning	2	0.36%
Pesticide poisoning	3	0.55%
Antidepressant poisoning	1	0.18%
Carbon monoxide poisoning	1	0.18%

A total of 388 patients underwent gastric lavage treatment, accounting for 70.5% of the overall cohort. The proportion of patients receiving gastric lavage decreased significantly from 2020 to 2022 (see Table 5). According to the “Chinese Expert Consensus on the Diagnosis and Treatment of Acute Poisoning,” gastric lavage should ideally be performed as early as possible—preferably within 1 h after poisoning. For certain toxins or in patients with delayed gastric emptying, this window may be extended to 6 h (9). In this study, time records from ingestion to gastric lavage were available for 371 patients. The proportion of patients who received gastric lavage within 6 h declined from 84.4% in 2020 to 68.0% in 2022 (*p* < 0.01). Similarly, the proportion of patients receiving gastric lavage beyond 6 h also decreased, from 57.1% in 2020 to 30.4% in 2022 (*p* < 0.05). Although the use of gastric lavage at our hospital has declined annually, its application rate remains higher than t developed countries. Moving forward, it is essential to strictly adhere to evidence-based indications and optimize timing criteria for gastric lavage in acute poisoning cases, ensuring its appropriate and judicious use.

**Table 5:** Comparison of gastric lavage characteristics of patients with gastric lavage from 2020 to 2022.

Variable	2020	2021	2022	*p*
Poisoning cases	150	171	229	<0.001^a^
Gastric lavage cases	120 (80.0%)	130 (76.0%)	138 (60.3%)	
Cases without gastric lavage	30 (20.0%)	41 (24.0%)	91 (39.7%)	
Poisoning cases without recorded time	7	6	11	
Gastric lavage cases without recorded time	5	5	7	
Poisoning cases with recorded time	143	165	218	
Gastric lavage cases with recorded time	115	125	131	
Gastric lavage cases within 6 h	103 (84.4%)	100 (81.3%)	117 (68.0%)	<0.01^a^
Cases without gastric lavage within 6 h	19 (15.6%)	23 (18.7%)	55 (32.0%)	
Gastric lavage cases after 6 h	12 (57.1%)	25 (59.5%)	14 (30.4%)	<0.05^a^
Cases without gastric lavage after 6 h	9 (42.9%)	17 (40.5%)	32 (69.6%)	

a Pearson chi square test.

## 4 Discussion

This study examined the demographic characteristics, risk factors, clinical profiles, treatments approaches, and outcomes of acute poisoning cases in the hospital’s emergency department between 2020 and 2022, encompassing all age groups. It represents the first comprehensive data collection on acute intoxication in the region. The results reveal that drug poisoning is the most common type of acute intoxication, particularly among young women, with a significant proportion of cases involving antidepressants and antipsychotics. These findings align with previous studies in other regions (1, 10). However, our study also identifies unique patterns, such as the higher incidence of poisoning on Mondays and Thursdays, which may be linked to occupational stress dynamics, particularly among individuals in high-pressure professional environments.

### 4.1 Gender differences in acute poisoning

Our study identified significant gender disparities in the epidemiology of acute poisoning, consistent with previous reports in the literature (11, 12). These differences are not only demographic but also reflect distinct patterns of toxin exposure, intent, and clinical presentation. The predominance of female patients (72.36%) and their younger median age (20 years vs. 25 for males) suggest that sociocultural and psychological factors play a key role in poisoning incidents involving women. However, the study from Iran reported a median age of 26 years for female patients involved in poisoning incidents. This difference suggests that the age distribution of poisoning cases may vary significantly across different cultural contexts (13). Drug intoxication was the most common form of poisoning among females (86.9%), compared to a lower proportion in males (60.5%). In contrast, males were more likely to be involved in pesticide, gas, or compound poisoning—likely reflecting occupational and behavioral risk differences. These findings align with global trends showing a higher prevalence of drug-related poisoning among women, consistent with previous studies (14). The huge association between female poisoning cases and suicidal intent is particularly striking. With 84.7% of female poisoning cases linked to suicide attempts-compared to 63.8% among males. These data highlight intoxication as a prevalent yet preventable method of self-harm among women. Compared to males, who often choose more immediately lethal methods such as hanging or falling (15). Studies from Europe and other Asian countries corroborates these findings, reporting similar gender imbalances in intentional drug poisoning (16–18).

From a public health perspective, these findings call for targeted interventions aimed at high-risk groups, especially young women. Integrating mental health screening into primary care and emergency settings can help identify at-risk individuals before poisoning occurs (13, 19). In regions where pesticide poisoning remains prevalent among men, stricter regulations and education on safe storage and use could significantly reduce mortality. Understanding gender differences in acute poisoning can provide actionable insights for healthcare providers, mental health professionals, and policymakers.

### 4.2 Temporal distribution of poisoning cases: weekly and seasonal trends

Our research reveals distinct temporal patterns in poisoning incidents, with a higher frequency observed on Mondays and Thursdays compared to weekends. These results were different from what we expected. Before the study, we thought there would be more poisoning cases on weekends. This was because weekends are often linked to more drinking and social activities, which can lead to alcohol poisoning. However, in this study, we did not include cases of pure alcohol intoxication, which may explain why we saw more cases on weekdays instead. By removing alcohol-related cases, we focused on other types of poisoning that may be related to stress, mental health, or self-harm. These factors are more common during the workweek, especially on Mondays and Thursdays (20, 21). Mondays and Thursdays often mark periods of heightened workload and responsibility, especially for young and middle-aged professionals navigating competitive environments. While weekends may provide temporary relief from these stressors, their recurrence at the beginning of the week could trigger emotional distress. Furthermore, mental health conditions, especially depression, are more commonly reported on weekdays. Such conditions may exacerbate symptoms and increase the risk of self-harm or suicide. These conditions may intensify symptoms and increase the risk of self-harm or suicide (22, 23). The discrepancy between the 2020 data and the three-year aggregate data is primarily attributable to 2020 being the first year of the COVID-19 outbreak, during which home quarantine measures led to significantly reduced human activities in specific periods, which in turn resulted in lower recorded case volumes.

Research has consistently shown a seasonal pattern in drug-related poisoning cases, with a peak during spring. One study reported that 27.16% of all poisoning incidents occurred in this season (24). This finding corroborated by our data, which identified April as the month with the highest incidence of acute poisoning. This aligns with broader evidence linking certain times of the year to elevated risks of self-harm, particularly through drug overdose. The majority of acute poisoning cases at our hospital involved individuals with a history of major depression who attempted suicide through drug overdose (25). This highlights the strong link between mood disorders and intentional self-poisoning, especially among young and middle-aged adults. Seasonal stress, poor coping mechanisms, and inadequate mental health support may drive the increase in poisoning cases during this period.

These findings highlight the importance of integrating temporal trends into public health planning and clinical preparedness, particularly for high-risk periods such as Mondays, Thursdays, and the spring season. Targeted interventions, including enhanced mental health screening, timely crisis intervention and public awareness campaigns, can help reduce the burden of intoxication and prevent future incidents of self-harm.

### 4.3 Patterns of intoxication

Among all reported intoxication cases, 85.45% involved drug intoxication. The most frequently encountered substances were antipsychotics, antidepressants, and benzodiazepines, which together accounted for a substantial 74.88% of all drug-related poisonings. Other commonly reported agents included antipyretics, analgesics, antibiotics, and antihistamines—often easily accessible medications. In the realm of non-pharmaceutical intoxications, gas poisoning represented approximately 6.18% of all cases, with carbon monoxide being the primary agent. Pesticide poisoning constituted 3.82%, predominantly involving organophosphates, rodenticides, and herbicides—substances widely used in agricultural practices. Additionally, compound poisoning, often resulting from exposure to household cleaning agents or industrial chemicals, made up 3.09% of the total cases. These findings reflect a distinct pattern compared to those observed in western countries, particularly the United States, where the leading causes of acute poisoning include analgesics, household cleaning products, antidepressants, cosmetics/personal care items, and cardiovascular drugs. In the U.S., opioid abuse remains a national crisis, with fentanyl playing a central role. According to the Centers for Disease Control and Prevention (CDC), there were 220,000 overdose deaths in 2022, with 70% linked to fentanyl—a substance that is strictly controlled in China (26). In contrast, while fentanyl abuse is relatively rare in China, emerging trends show increasing misuse of newly regulated psychoactive substances, such as zolpidem, and potentially addictive but currently unregulated compounds like dextromethorphan and compound diphenoxylate (27). This evolving landscape calls for timely policy updates and enhanced monitoring systems. As new psychoactive substances gain popularity, regulators must act swiftly to monitor and control their distribution. This includes updating legal frameworks to regulate emerging substances such as zolpidem and dextromethorphan (28).

### 4.4 The role of gastric lavage in the treatment of acute poisoning

Gastric lavage was historically considered a cornerstone for toxin removal; however, its routine use has been significantly restricted due to potential complications such as gastrointestinal bleeding and aspiration pneumonia. According to guidelines from the American Association of Poison Control Centers (AAPCC) and a multicenter prospective study, only 45.8% of cases met the appropriate criteria for gastric lavage. Current clinical protocols emphasize individualized patient assessment and prioritize the use of activated charcoal in combination with targeted blood purification therapies (29). Significant regional variations exist in the application of gastric lavage. Appropriate use rates are lower in Eastern Europe, Southern Europe, the Eastern Mediterranean region, and South America compared to North America, Western Europe, Northern Europe, and the Western Pacific region (30). The 2023 Annual Report of the American Association of Poison Control Centers indicates that only 0.02% of poisoning patients in the United States underwent gastric lavage (26). According to a 2021 German study, just 10.9% of patients received a primary detoxification intervention (31). These data suggest that as medical knowledge and technology advance, gastric lavage is gradually being replaced by safer and more effective alternatives.

Unlike the predominantly drug-related poisoning cases seen in Europe and the United States, pesticide poisoning has historically been the leading cause of acute intoxication in China. As a result, gastric lavage remains a commonly used method for toxin removal (32). According to the Chinese Expert Consensus on Diagnosis and Treatment of Acute Poisoning, gastric lavage should ideally be performed within 1 h after ingestion, although this window can be extended to 4–6 h for certain toxins or in patients with delayed gastric emptying. For pesticide poisoning, such as organophosphate or paraquat intoxication, an aggressive approach involving early and thorough lavage is recommended, whereas a more conservative strategy is advised for drug overdoses (9). Our institution has observed a steady decline in the use of gastric lavage for acute poisoning. The overall proportion of patients receiving this procedure dropped from 80.0% in 2020 to 60.62% in 2022—a statistically significant decrease. Similarly, the proportion of patients treated within 6 h fell from 87.4 to 67.3%, and those treated beyond 6 h decreased from 57.1 to 40.9%. Despite this downward trend, gastric lavage remains more frequently used in our setting compared to many developed countries in Europe and North America. The differences between China and other countries show variations in poisoning patterns. These differences also reflect how public health policies and social factors affect poisoning rates and treatment choices. Comparing practices across regions can help improve treatment strategies and patient care. National and regional medical authorities should encourage the adoption of modern detoxification methods such as activated charcoal and blood purification therapies, especially for drug-related poisoning.

## 5 Conclusion

This study investigated the demographic characteristics, risk factors, clinical profiles, treatment approaches, and outcomes of acute poisoning cases managed at our hospital’s emergency center between 2020 and 2022. Our findings indicate that drug poisoning is the most prevalent form of acute poisoning in this setting. Female patients not only accounted for a larger proportion of total poisoning cases, drug-related poisonings, and suicide attempts, but also tended to be younger than their male counterparts. Notably, we observed significant temporal and seasonal variations in the incidence of acute poisoning. We have analyzed the potential drivers behind these gender-based and time-based differences, considering sociocultural, behavioral, and environmental factors.

The results highlight the distinct epidemiological profile of acute poisoning in Hangzhou, underscoring the need for tailored public health interventions. Comparative analyses with poisoning patterns and policy responses in other countries reveal how differences in pharmaceutical regulation, cultural norms, and healthcare systems contribute to variations in poisoning trends. Moving forward, a comprehensive and integrated strategy—combining clinical readiness, mental health services, public education, and innovative policy initiatives—is crucial to mitigating the burden of poisoning and improving patient outcomes.

This single-center retrospective study has several limitations. As the hospital is located in western Hangzhou rather than the city center, the findings may not fully represent the overall epidemiology of acute poisoning in the region. Incomplete case records and a relatively short three-year study period further limit the generalizability of the results. Multicenter prospective studies are needed to better understand the broader patterns and risk factors of acute poisoning across diverse populations.

## Data Availability

The original contributions presented in the study are included in the article/supplementary material, further inquiries can be directed to the corresponding author.
